# Oritavancin Multiple Dosing for Complex Infections: A Pharmacokinetic/Pharmacodynamic Simulation Study

**DOI:** 10.3390/pharmaceutics18050628

**Published:** 2026-05-20

**Authors:** Ana Alarcia-Lacalle, Miguel Ángel Morán-Rodríguez, Laura Morata, Arantxa Isla, Andrés Canut-Blasco, Alicia Rodríguez-Gascón

**Affiliations:** 1 Pharmacokinetic, Nanotechnology and Gene Therapy Group (PharmaNanoGene), Faculty of Pharmacy, Lascaray Research Centre, University of the Basque Country EHU, Paseo de la Universidad n° 7, 01006 Vitoria-Gasteiz, Spain; ana.alarcia@ehu.eus (A.A.-L.); arantxa.isla@ehu.eus (A.I.); 2 Infectious Disease Division, Araba University Hospital, Osakidetza Basque Health Service, Francisco Leandro de Viana Kalea, 01009 Vitoria-Gasteiz, Spain; miguelangel.moranrodriguez@osakidetza.eus; 3 Microbiology, Infectious Disease, Antimicrobial Agents, and Gene Therapy, Bioaraba Health Research Institute, Jose Atxotegi Kalea s/n, 01009 Vitoria-Gasteiz, Spain; andres.canutblasco@osakidetza.eus; 4 Department of Infectious Diseases, Hospital Clínic of Barcelona, University of Barcelona, Carrer de Villarroel, 170, 08036 Barcelona, Spain; lmorata@clinic.cat; 5 Microbiology Service, Araba University Hospital, Osakidetza Basque Health Service, Francisco Leandro de Viana Kalea, 01009 Vitoria-Gasteiz, Spain

**Keywords:** oritavancin, complex infection, PK/PD, *f*C_min_ > MIC, AUC/MIC, Monte Carlo simulation

## Abstract

**Background/Objectives:** Oritavancin therapy for complex infections remains challenging due to the lack of well-established dosing regimens. The objective of this work was to apply PK/PD modeling and Monte Carlo simulation considering different PK/PD targets to identify multiple-dosing regimens that may ensure effective concentrations of oritavancin for the treatment of long-term infections. **Methods:** Plasma concentration–time profiles were simulated for different regimens (single dose of 1200 mg, 1200 mg followed by 800 mg every 7 days (q7d), 1200 mg followed by 800 mg q10d, 1200 mg q7d, 1200 mg q10d, 1200 mg q14d, 1200 mg q21d, and 1200 mg followed by 1200 mg on day 8, then 1200 mg q14d), and the probability of target attainment (PTA), indicative of treatment success, was estimated. **Results:** All dosing regimens provided probabilities of target attainment of 100% up to MICs of 0.5 mg/L when AUC_0–24_/MIC and C_max_/MIC were applied. Considering AUC_0–72_/MIC, the regimens would be adequate up to an MIC of 0.125 mg/L. For *f*C_min_ > MIC, all except 1200 mg q21d were adequate for an MIC of 0.125 mg/L, and 1200 mg day 1 + 800 mg q7d and 1200 mg q10d may be useful to treat infections due to bacteria with an MIC of 0.25 mg/L. **Conclusions:** More studies involving patients with complex infections are needed to better stablish the relationships among plasma concentrations, MIC values, and clinical outcomes. *f*C_min_ > MIC should be investigated as a potential PK/PD target for the treatment of these infections with oritavancin.

## 1. Introduction

Oritavancin is a long-acting lipoglycopeptide antibiotic with potent activity against a broad spectrum of Gram-positive bacteria [1,2,3]. It was approved in 2014 by the US Food and Drug Administration (FDA) and in 2015 by the European Medicines Agency (EMA) for the treatment of acute bacterial skin and skin structure infections (ABSSSIs) caused by susceptible Gram-positive microorganisms. The standard approved regimen for this indication consists of a single 1200 mg intravenous dose, traditionally administered over a 3 h infusion, although a newer formulation (Kimyrsa^®^) allows the same dose to be infused over 1 h [4]. Oritavancin displays linear pharmacokinetics over the studied dose range and it is characterized by rapid and extensive tissue distribution, concentration-dependent bactericidal activity, and a notably prolonged terminal half-life, reported to range from approximately 245 to 393 h [5]. This extended half-life renders oritavancin a theoretically attractive option for the management of infections requiring sustained antimicrobial exposure [6].

In addition to its approved indication for ABSSSIs, oritavancin has been increasingly investigated and adopted in clinical practice for the treatment of more complex and deep-seated Gram-positive infections, often on an off-label basis. These include challenging conditions such as osteomyelitis, prosthetic joint infections, bacteremia, and infective endocarditis, as well as infections involving vancomycin-resistant *Enterococcus* spp., against which oritavancin demonstrates notable activity [3,6]. However, the evidence supporting these off-label applications is largely limited to case reports, small case series, and observational studies [7,8].

A major challenge in using oritavancin for extended therapy beyond the approved single dose is the absence of well-established dosing regimens for multi-dose administration. Observational data describe varied multi-dose schemes, often involving initial 1200 mg doses followed by repeated doses of 800 mg or 1200 mg at weekly or longer intervals, such as every 14 or 28 days [9]. While pharmacokinetic (PK) modeling has suggested that a two-dose regimen (e.g., 1200 mg followed by 800 mg a week later) may prolong therapeutic exposures [10], PK data for prolonged multi-dose regimens are still limited.

Oritavancin presents concentration-dependent activity; it has been shown to exhibit rapid bactericidal activity against a wide range of Gram-positive bacteria, including those with reduced susceptibility to vancomycin [1,9]. The application of pharmacokinetic/pharmacodynamic (PK/PD) principles to guide oritavancin therapy is challenging due to the absence of clearly defined PK/PD targets. While the area under the concentration–time curve divided by the minimum inhibitory concentration (AUC/MIC) has been identified as the PK/PD parameter best correlated with oritavancin’s efficacy, and nonclinical models support specific AUC/MIC targets for bacteriostasis or bacterial reduction, these relationships and targets were primarily established in the context of single-dose therapy for ABSSSIs [11]. The applicability of these targets to multi-dose regimens or to infections in sites with potentially different drug penetration (e.g., bone, central nervous system) remains uncertain. This uncertainty highlights the urgent need for further research to define appropriate multi-dose regimens, establish reliable PK/PD targets for different types of infections, and potentially guide therapeutic drug monitoring (TDM) to optimize the use of oritavancin beyond its currently approved indication. The objective of this work was to apply PK/PD modeling and Monte Carlo simulation considering different PK/PD targets to identify multiple-dosing regimens that may ensure effective concentrations of oritavancin for the treatment of long-term infections.

## 2. Materials and Methods

Different simulations were conducted to evaluate the predicted oritavancin concentrations resulting from different dosing regimens, including a single dose of 1200 mg, 1200 mg on day 1 followed by 800 mg every 7 days (q7d), 1200 mg on day 1 followed by 800 mg every 10 days (q10d), 1200 mg q7d, 1200 mg q10d, 1200 mg every 14 days (q14d), 1200 mg every 21 days (q21d), and 1200 mg on day 1 followed by 1200 mg on day 8, then 1200 mg q14d. All regimens were simulated as intravenous infusions administered over 3 h.

The population pharmacokinetic model used for the simulations was based on a model previously published by Rubino et al. [5], which included data from 297 patients receiving a single 1200 mg intravenous dose of oritavancin infused over 3 h. Simulations of total plasma concentrations for 10,000 hypothetical patients were performed using NONMEM (version 7.5; Icon Development Solutions, Hanover, MD, USA) within the Pirana (version 2.9.7; Pirana Software & Consulting, San Francisco, CA, USA) workbench. The interindividual variability for the pharmacokinetic parameters described in the three-compartment model [5] was applied, along with additive and proportional residual errors. The results from NONMEM simulations, including data formatting, graphical outputs, and statistical summaries, were processed using R (version 4.4.2; R Foundation for Statistical Computing, Vienna, Austria) in RStudio (2024.12.1+563; Posit, Boston, MA, USA). In order to corroborate the model consistency, we estimated some pharmacokinetic parameters from simulated concentrations and compared them to those obtained by the original model [5] and by Rose et al. [10]. A statistical comparison was performed using a two-sided *t*-test.

From plasma concentration simulations, the probability of reaching the targeted exposure or the probability of target attainment (PTA) was estimated. The PTA corresponds to the percentage of simulated patients with an estimated PK/PD index equal to or higher than the value related to the efficacy of the antibiotic against a pathogen with a certain MIC [12]. An effective dosing regimen was considered as one achieving a PTA ≥ 90% [13]. Given the absence of a broadly recognized pharmacokinetic/pharmacodynamic (PK/PD) target for oritavancin, various PK/PD targets that have been suggested to correlate with efficacy were taken into account. On the one hand, the maximum free concentration of the drug in serum to the MIC ratio (*f*C_max_/MIC > 14) was considered [14]. On the other hand, the ratio between the area under the concentration–time curve from 0 to 24 h (AUC_0–24_) and the MIC of the specific microorganism causing the infection higher than 100 (AUC_0–24_/MIC > 100) [1,15] and the ratio between the area under the concentration–time curve from 0 to 72 h (AUC_0–72_) and the MIC higher than 4581 (AUC_0–72_/MIC > 4581) [16] were explored. Additionally, we calculated the probability of the total minimum concentration (C_min_) exceeding a concentration threshold of 2 mg/L or 3 mg/L [7]. Finally, the probability of the free C_min_ exceeding the European Committee on Antimicrobial Susceptibility Testing (EUCAST) clinical breakpoint or the MIC required to inhibit 90% of isolates (MIC_90_) was also calculated (*f*C_min_ > MIC). The unbound fraction (0.13) was obtained from the literature [17].

To estimate the PTA values, the MIC_90_ of the microorganisms related to complex infections was considered, which was obtained from Pfaller et al. (includes unique clinical isolates collected in 2010–2019 in European medical centers, SENTRY Program) [18]. Clinical breakpoints reported by the EUCAST were also considered [19]. Table 1 shows the microorganisms involved, the MIC_90_ and the clinical breakpoints used for this study. Additionally, we considered the MIC of 0.5 mg/L to estimate the PTA, since, although much less frequent, isolates with this MIC may exist.

## 3. Results

**Figure 1:**
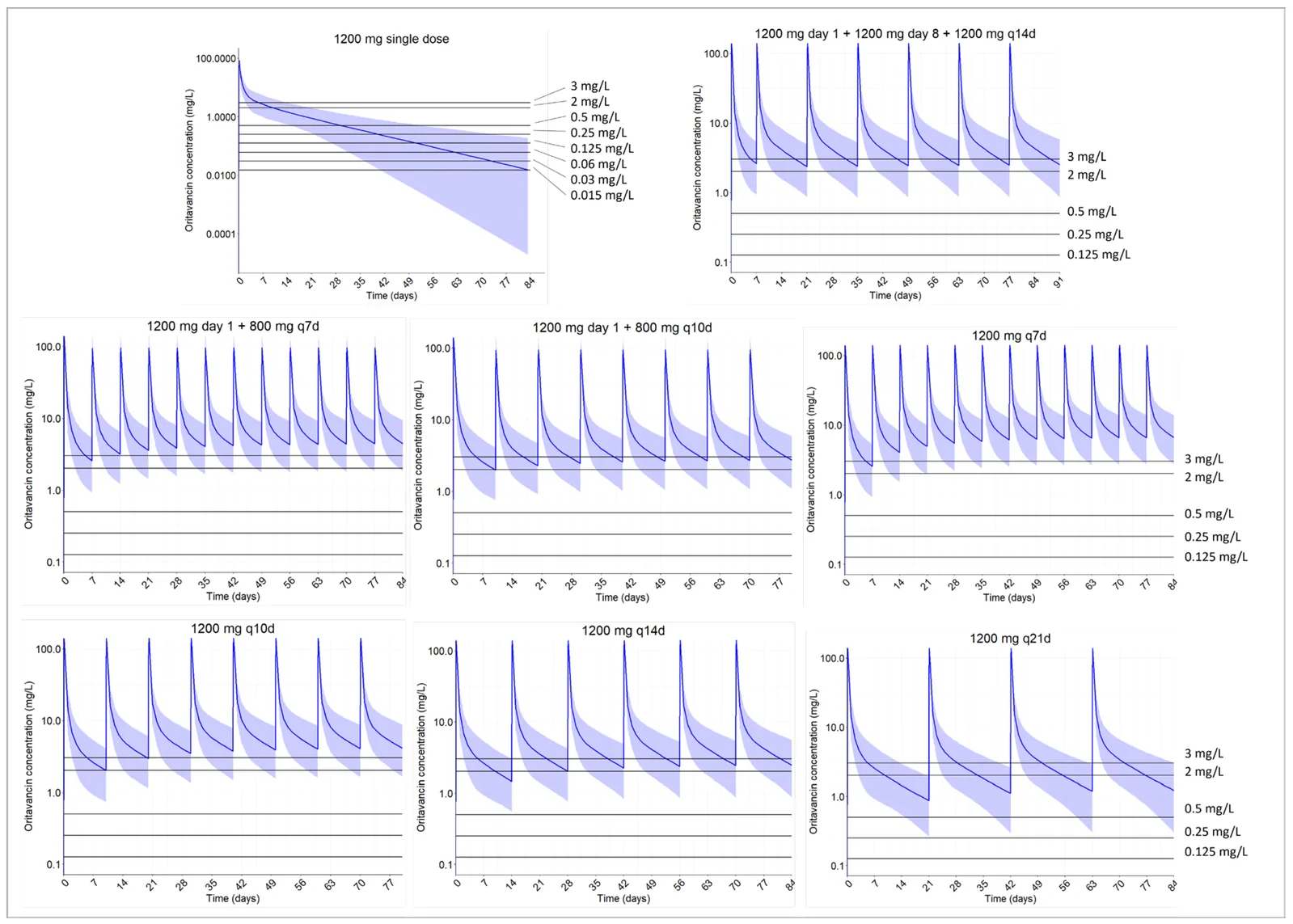
Simulated oritavancin plasma concentration–time profiles for different dosage regimens. The blue line represents the median of the simulated oritavancin plasma concentrations, and the shaded area represents the simulation-based 95% confidence interval for the median. Horizontal dotted lines indicate the concentration values used for the estimation of PK/PD target attainment (concentration thresholds of 2 mg/L and 3 mg/L, and representative MIC values).

Figure 1 shows the simulated plasma concentrations of oritavancin over time for the different dosage regimens studied. Except with the single dose, the total drug concentrations were always higher than 0.25 mg/L from the first oritavancin administration.

Table 2 features the comparison of the pharmacokinetic parameters of oritavancin achieved by simulating the plasma concentrations after a single dose of 1200 mg with those reported by Rubino et al. [5] and Rose et al. [10]. As shown in the table, only slight differences were detected, which confirms that our simulation code accurately reproduces the original model’s output.

Once the validity of the simulations was corroborated, the estimation of the probability of attaining the different PK/PD targets was carried out. For *f*C_max_/MIC > 14 and AUC_0–24_/MIC > 100 as PK/PD indexes related to efficacy, the PTA was always 100%, even for an MIC of 0.5 mg/L.

Table 3 shows the PTA considering the PK/PD index AUC_0–72_/MIC > 4581 for different MIC values. Regardless of the dosage regimen, the PTA was 100% up to MICs of 0.125 mg/L. For a 0.25 mg/L MIC value (EUCAST clinical breakpoint and MIC_90_ for β-hemolytic streptococci), 1200 mg (single or multiple dose) provided a PTA close to, but lower than, 90%. Since the plasma concentrations at steady state depend on the maintenance dose and not on the first dose, the two dose regimens that included repeated administrations of 800 mg provided a lower PTA at steady state: 68% with 1200 mg on day 1 + 800 mg q7d, and 50% with 1200 mg on day 1 + 800 mg q10d. For 0.5 mg/L, the PTA was ≤1% with all the dosing regimens studied.

In Table 4, the probability that the oritavancin minimum concentrations (C_min_) at steady state are higher than 2 mg/L or 3 mg/L is presented, as well as the probability that the C_min_ is higher than representative MIC values. For the single dose of 1200 mg, the PTA values were estimated at the times when the next doses were administered in the multiple-dose regimens. The PK simulations revealed that, as expected, the administration of repeated doses increased the probability of achieving C_min_ > 2 mg/L and C_min_ > 3 mg/L. At steady state, a probability of achieving C_min_ > 2 mg/L greater than 90% was observed only with the administration of 1200 mg on day 1 + 800 mg q7d, 1200 mg q7d, or 1200 mg q10d. Increasing the dosing interval reduced the probability of target attainment. With 1200 mg on day 1 + 800 mg q10d, the PTA decreased to 73%. For the higher target of C_min_ > 3 mg/L, only the 1200 mg q7d regimen achieved a probability above 90%, and as observed for C_min_ > 2 mg/L, extending the dosing interval led to lower PTA values. The administration of 800 mg after the 1200 mg initial dose also led to lower PTA values (80% or 44% if the dose of 800 mg was administered q7d or q10d, respectively). Considering the MIC_90_ of *E. faecium* (0.015 mg/L), the MIC_90_ of *E. faecalis* (0.03 mg/L) and the MIC_90_ of *S. aureus* and coagulase-negative staphylococci (0.06 mg/L), with the administration of a 1200 mg single dose, the probability of a *f*C_min_ > MIC higher than 95% was maintained for at least 21 days; however, it lowered to 14 and 10 days for MIC values of 0.06 mg/L and 0.125 mg/L, respectively. For multiple-dose regimens, in all cases except 1200 mg q21d, there was high probability (>90%) of reaching *f*C_min_ > 0.125 mg/L, and only with 1200 mg on day 1 + 800mg q7d, 1200 mg q7d and 1200 mg q10d, the probability of reaching *f*C_min_ > 0.25 mg/L was higher than 90%. None of the dosage regimens studied resulted in the PTA reaching values equal to or greater than 90%, and only 1200 mg q7d provided a PTA higher than 80%.

## 4. Discussion

Resistant Gram-positive infections such as infective endocarditis and osteoarticular infections, particularly those involving prosthetic material, pose significant therapeutic challenges. These conditions often necessitate prolonged intravenous antibiotic therapy due to the lack of effective oral alternatives, increasing the risk of catheter-related complications, healthcare resource utilization, and overall costs. The prolonged terminal elimination half-life and prolonged plasma levels for extended periods after administration [5,9], together with its potent in vitro activity against Gram-positive pathogens, make oritavancin a potential therapeutic option for infections requiring long-lasting antimicrobial exposure. In real-world clinical settings, different multiple-dose regimens have been used with success in treating a variety of infections besides ABSSSIs, and with a good safety profile [9,20]. However, there is no consensus on the optimal dosing strategy for managing these types of infections, in terms of both the dose and the interval.

Although the role of TDM has been highlighted for guiding and individualizing oritavancin administration for complex infections, including prosthetic joint infections and osteomyelitis, there is an important lack of information about oritavancin TDM. Buonomo et al. [7] and Bongiovanni et al. [8] published case reports with the C_min_ levels measured in patients after administering multiple doses of oritavancin, and they related them with the efficacy; unfortunately, only three patients were included, one with a methicillin-resistant *Staphylococcus epidermidis* infection and two with MRSA infections. All three patients achieved clinical cure. Buonomo et al. suggested that C_min_ > 3 mg/L may be associated with therapeutic effectiveness; however, our simulations showed that the probability of achieving a C_min_ higher than 3 mg/L with the dosing regimens administered to the patients was below 80%. In fact, among all the dosages we evaluated, only 1200 mg q7d ensured a probability of C_min_ > 3 mg/L higher than 90%. Based on these results, it remains unclear whether C_min_ > 3 mg/L or even C_min_ > 2 mg/L are appropriate targets for TDM, as lower plasma concentrations may also be sufficient to achieve clinical efficacy, particularly considering the low MIC values of the clinical isolates (Table 1). These targets reveal the weakness of not relating to the MIC of the microorganism responsible for the infection. This may increase the risk of overdosing in an attempt to achieve the objective. Therefore, additional studies are needed to confirm the usefulness of these targets for TDM, as well as to establish whether they may be an alternative for predicting the efficacy to AUC/MIC, the key PK/PD index for clinical efficacy in humans [9].

Despite the fact that TDM may offer valuable opportunities to enhance the treatment efficacy and safety, a PK/PD analysis is also a valuable approach for optimizing antibiotic dosing regimens, aiming to enhance the therapeutic efficacy while minimizing the emergence of multidrug-resistant pathogens [21,22]. PK/PD considers not only the drug exposure, but also the susceptibility of the causative microorganism. Unfortunately, there is a lack of consensus on the optimal PK/PD targets that predict clinical efficacy for oritavancin, particularly when it is administered in multiple-dose regimens. In fact, different PK/PD targets have been proposed, primarily applied to the treatment of ABSSSIs, including AUC_0–24_/MIC > 100 [1,15] and AUC_0–72_/MIC > 4581 (proposed as relevant for bactericidal activity in deep-seated or difficult-to-treat infections [16]). In a murine model of an *S. aureus* infection, Boylan et al. [23] found a high correlation of the AUC, C_max_, and T > MIC with efficacy, although C_max_ appeared to have the best correlation. However, by using a rabbit experimental endocarditis due to vancomycin-susceptible or -resistant *E. faecalis*, other authors found that increasing the peak serum levels and AUC did not improve the in vivo activity of the antibiotic [24]. A *f*C_max_/MIC ratio of approximately 14 has been associated with a near-maximal effect (~1 to 1.5 log reduction in bacterial density) [14]. Considering this target, all dosing regimens provided a probability of target attainment of 100%, even for the MIC of 0.5 mg/L. Similar results were obtained with AUC_0–24_/MIC > 100. Therefore, these targets did not prove useful in discriminating among dosing regimens and MIC values, at least up to an MIC of 0.5 mg/L. Regarding AUC_0–72_/MIC > 4581, all the dosing regimens were successful up to an MIC of 0.125 mg/L (PTA of 100%). For the MIC of 0.5 mg/L, these dosages failed to attain the PK/PD target.

Other targets aim to ensure that the free drug concentrations remain above the MIC throughout the entire dosing interval (only the unbound drug is able to access the tissue, and therefore, the unbound antibiotic at the infection site is responsible for the effect [25]). For instance, in rabbit experimental endocarditis due to vancomycin-susceptible or -resistant *E. faecalis*, Lefort et al. [24] suggested that oritavancin activity seems to be more time-dependent than dose-dependent. Therefore, we considered different targets based on the probability that *f*C_min_ exceeds either the MIC_90_ or the EUCAST clinical breakpoints of the relevant microorganisms commonly involved in complex infections (Table 4). This approach is consistent with previous findings showing good correlation of the time during which free plasma drug concentrations exceed the MIC (%*f*T _> MIC_) with efficacy [23]. Although a %*f*T _> MIC_ ranging from 22% to 50% has been proposed [14], since oritavancin dosing regimens may be useful for the treatment of complex infections, and in order to be more conservative, we estimated the probability that *f*C_min_ is over the MIC, that is, %*f*T _> MIC_ of 100%. This target, considering both the MIC values and *f*C_min_, allowed us to detect differences in the probability of success of the dosage regimens. For MIC values up to 0.125 mg/L, with all dosage regimens except 1200 mg q21d, the probability that *f*C_min_ > MIC, and therefore, the probability of target success, was 90% or higher. When the MIC was 0.25 mg/L, the dosing regimens that provided a high probability of success (>90%) were 1200 mg on day 1 + 800 mg q7d, 1200 mg q7d, and 1200 mg q10d. For an MIC of 0.5 mg/L, 100 mg q7d provided a PTA lower than, but close to, 90%; all of the other dosing regimens were insufficient. These findings are consistent with previous studies reporting that multiple doses of oritavancin are associated with high clinical success rates (up to 90%) in patients with bone and joint infections, including those caused by MRSA [2,9,26,27].

In spite of the fact that our findings suggest that *f*C_min_ > MIC could help guide oritavancin dosing, clinical validation is needed to confirm this hypothesis. One of the main advantages of *f*C_min_ is its greater practicality for routine application in TDM, compared to targets such AUC_0–72_/MIC > 4581, which require individual estimation of drug clearance to calculate the AUC_0–72_. In contrast, *f*C_min_ can be more easily measured in clinical settings, even though the unbound fraction is typically derived from literature values. Moreover, given that the lower limit of quantification of the currently available techniques for measuring oritavancin in plasma is 12.5 ng/mL (0.0125 mg/L) [5,28,29], it is highly unlikely that C_min_ values would fall below this threshold, even for the regimens that provide the lowest C_min_ values: 1200 mg on day 1 + 800 mg q7d, 1200 mg q21d, and 1200 mg on day 1 + 1200 mg on day 8 + 1200 mg q14d (Figure 1). It is important to take into account that, for infections such as osteomyelitis or prosthetic joint infections, efficacy depends on the ability of the antibiotic to reach deep-seated infections, such as in the synovial fluid or bone. In an in vivo model of rabbits [28], oritavancin showed a good penetration to the bone (the tissue-to-serum AUC_0–168_ ratio was 1.7 and 3.1 in the bone matrix and bone marrow, respectively). In that study, the results were based on the total drug concentrations, and since the active fraction in bone remains to be elucidated, the PK/PD relationship of oritavancin in bone and other tissues should be evaluated cautiously. In line with this, the proposed dose regimens should be confirmed in clinical studies, with a measurement of oritavancin in diseased bone tissues, if feasible. Another relevant consideration is the capacity of certain bacteria, including *S. aureus*, to form biofilms. Oritavancin has demonstrated activity against both planktonic and biofilm-embedded bacteria, a particularly relevant property given the contribution of biofilms to the chronicity of bone and periprosthetic joint infections [30]; however, attaining *f*C_min_ > MIC, while necessary, may not be sufficient to achieve eradication in deep-seated bone infections.

Some of the dosing regimens with a high probability of treatment success involved the administration of repeated doses of 800 mg, either every 7 days or every 10 days. In those cases, an important consideration is the difficulty of dose fractionation when using the Kimyrsa formulation, developed to simplify the preparation of the solution for infusion. Kimyrsa is packaged as a single vial containing 1200 mg of oritavancin to be reconstituted with 250 mL of infusion solution and administered intravenously over 1 h [4], contrary to the first approved formulation, which is packaged in three single-use vials to be reconstituted with 1000 mL of infusion solution. In spite of the clear advantages of Kimyrsa over the first approved formulation, the adaptation of the antibiotic presentation to dosing requirements should also be considered.

Despite the usefulness of the PK/PD analysis in optimizing oritavancin-dosing regimens, its clinical application is limited by the difficulty in obtaining reliable MIC values. In fact, oritavancin susceptibility testing is not routinely included in standard antimicrobial panels in most clinical microbiology laboratories. Instead, it requires specialized broth microdilution (BMD) methods, often with supplemental additives, and it is usually performed only in reference laboratories or selected academic centers [31]. In this context, additional efforts to implement clinical microbiology laboratories for oritavancin testing should be made.

This study presents some limitations that should be considered when interpreting the results. First, no population pharmacokinetic models of oritavancin are currently available for patients with infections other than ABSSSIs or after administration of multiple doses. Consequently, the simulations were based on the population PK model developed by Rubino et al. [5], which was built using data from patients receiving a single 1200 mg dose. Additionally, limited concentration data are available in the terminal elimination phase, which introduces uncertainty in the characterization of the late pharmacokinetic profile. According to this population model, a covariate analysis suggested that no dose adjustment is required for mild or moderate renal or hepatic impairment, body weight, age, diabetes or sex. However, due to the scarce number of studies describing the pharmacokinetics of oritavancin, particularly after multiple dosing, additional studies are needed to better estimate the inter-patient variability and to confirm the influence of covariates in the PK parameters. Moreover, we used a fixed value of the unbound fraction obtained from the literature; in this regard, studies investigating the variability in oritavancin protein binding would be helpful to refine our simulations. Second, we extrapolated the population model developed after the administration of a single dose to multiple dosing. Although the pharmacokinetics of oritavancin have been shown to be linear across a total dose range from 3.66 to 44.6 mg [32], no data on potential changes in clearance upon repeated administration, tissue accumulation, or enzyme induction/inhibition are available. Therefore, since this model has not been validated for multiple-dose regimens, the results must be interpreted with caution. Third, the population pharmacokinetic model was developed based on data from patients receiving the initially approved formulation. Given the similar pharmacokinetic profile of oritavancin with the two formulations, our results are also applicable to Kimyrsa, which, due to its administration-related advantages from the patient’s perspective, is expected to experience increased clinical use.

Although our study could help to select the most appropriate dose regimen of oritavancin, it is important to note that the results are based on simulations. Nevertheless, this work may be useful in the design of clinical studies to better establish the relationship between the PK/PD and the efficacy of oritavancin, and to identify the best PK/PD target. These studies will help to define dosing recommendations and to confirm the usefulness of *f*C_min_ > MIC to individualize the treatment with oritavancin and to apply model-informed precision dosing. This approach allows for personalized dosing, avoiding unnecessary overexposure to the antimicrobial agent and improving its cost-effectiveness. Another important issue is that, although a recent review study [9] confirmed that oritavancin is safe even when it is administered as multiple doses (only few or no adverse effects have been reported), the safety of the optimized dosages should also be confirmed.

## 5. Conclusions

According to our simulations, all the dosing regimens studied provided probabilities of target attainment of 100% up to MICs of 0.5 mg/L when AUC_0–24_/MIC and C_max_/MIC were applied. Considering AUC_0–72_/MIC, the regimens would be adequate up to an MIC of 0.125 mg/L. If we consider *f*C_min_ > MIC, all except 1200 mg q21d were adequate for an MIC of 0.125 mg/L, and 1200 mg on day 1 + 800 mg q7d or 1200 mg q10d may be useful to treat infections due to bacteria with an MIC of 0.25 mg/L. More studies involving patients with complex infections are needed to better stablish the relationships among plasma concentrations, MIC values, and clinical outcomes. *f*C_min_ > MIC should be investigated as a potential PK/PD target for the treatment of these infections with oritavancin.

## Figures and Tables

**Table 1:** MIC_90_ and clinical breakpoints of the microorganisms related to joint infections considered for the study, reported by Pfaller et al. (European medical centers, SENTRY Program) and EUCAST, respectively.

Microorganism	SENTRY Antimicrobial Surveillance Program (2010–2019) [18]		EUCAST Clinical Breakpoint (mg/L) [19]
	N	MIC_90_ (mg/L)	
*Enterococcus faecalis*	4219	0.03	n.r.
*Enterococcus faecium*	2713	0.015	n.r.
*Staphylococcus aureus*	25,203	0.06	0.125
Methicillin-susceptible *Staphylococcus aureus* (MSSA)	19,199	0.06	
Methicillin-resistant *Staphylococcus aureus* (MRSA)	6004	0.06	
Coagulase-negative staphylococci	4374	0.06	n.r.
Methicillin-susceptible	1565	0.06	
Methicillin-resistant	2809	0.06	
β-hemolytic streptococci	4263	0.25	0.25

N: number of isolates; n.r.: not reported.

**Table 2:** Comparison of pharmacokinetic parameters obtained after simulation of oritavancin concentrations with those reported by Rubino et al. and Rose et al. No significant differences were detected (*p* < 0.05, two-sided *t*-test). Values are median and 90% confidence interval.

Parameter	Our Simulation	Rubino et al. [5]	Rose et al. [10]
Dose	1200 mg sd	1200 mg sd	1200 mg on day 1 + 800 mg on day 8
C_max_ (mg/L)	142 (89–207)	135 (94–187)	133 (70–245)
AUC_0–24_ (mg h/L)	1062 (728–1492)	1050 (686–1720)	
AUC_0–48_ (mg h/L)	1298 (899–1828)	1310 (836–2160)	
AUC_0–72_ (mg h/L)	1433 (1005–2016)	1430 (910–2420)	1399 (778–2481)
AUC_0–576_ (mg h/L)	2386 (1588–3529)	2350 (1590–3750)	
AUC_0-∞_ (mg h/L)	2692 (1725–4165)	2640 (1590–3750)	
*f*AUC while above 0.12 mg/L (mg h/L)	394 (248–617)		353 (142–859)
C_min_ at day 29 (mg/L)	0.52 (0.09–1.31)		0.53 (0.02–2.46)

C_max_: maximum concentration; AUC: area under the concentration–time curve; *f*AUC: area under the free concentration–time curve; C_min_: minimum concentration; sd: single dose.

**Table 3:** Probability of target attainment (PTA) for different dose regimens of oritavancin and different MIC values, considering AUC_0–72_/MIC > 4581 as the PK/PD index related to efficacy. For an MIC of 0.25 mg/L or 0.5 mg/L, the PTA was calculated for the first dose and at steady state.

Dosage Regimen	Probability (%) AUC_0–72_/MIC > 4581							
	MIC of 0.015 mg/L ^a^	MIC of 0.03 mg/L ^b^	MIC of 0.06 mg/L ^c^	MIC of 0.125 mg/L ^d^	MIC of 0.25 mg/L ^e^		MIC of 0.5 mg/L	
					First Dose	Steady State	First Dose	Steady State
1200 mg single dose	100	100	100	100	85	-	1	-
1200 mg on day 1 + 800 mg q7d	100	100	100	100	85	68	1	1
1200 mg on day 1 + 800 mg q10d	100	100	100	100	85	50	1	0
1200 mg q7d	100	100	100	100	85	85	1	1
1200 mg q10d	100	100	100	100	85	85	1	1
1200 mg q14d	100	100	100	100	85	85	1	1
1200 mg q21d	100	100	100	100	85	85	1	1
1200 mg on day 1 + 1200 mg on day 8 + 1200 mg q14d	100	100	100	100	85	85	1	1

^a^: MIC_90_ for *E. faecium*; ^b^: MIC_90_ for *E. faecalis*; ^c^: MIC_90_ for *S. aureus* and coagulase-negative staphylococci; ^d^: EUCAST clinical breakpoint of *S. aureus*; ^e^: EUCAST clinical breakpoint and MIC_90_ for β-hemolytic streptococci. AUC_0–72_: area under the plasma concentration–time curve from 0 to 72 h; MIC: minimum inhibitory concentration. q7d: every 7 days; q10d: every 10 days; q14d: every 14 days; q21d: every 21 days.

**Table 4:** Probability of target attainment (PTA) expressed as the probability that oritavancin minimum concentrations at steady state are higher than 2 mg/L or 3 mg/L, and higher than representative MIC values. For a 1200 mg single dose, the PTA values were estimated at times when the next doses were administered in the multiple-dose regimens.

	Probability (%)							
	C_min_>		*f*C_min_>					
Dosage Regimen	2 mg/L	3 mg/L	0.015 mg/L ^a^	0.03 mg/L ^b^	0.06 mg/L ^c^	0.125 mg/L ^d^	0.25 mg/L ^e^	0.5 mg/L
1200 mg single dose:								
at 168 h (7 days)	68	38	100	100	100	95	71	21
at 240 h (10 days)	50	20	100	100	99	90	53	7
at 336 h (14 days)	27	6	100	100	98	80	30	1
at 504 h (21 days)	5	0	99	97	86	46	6	0
1200 mg on day 1 + 800 mg q7d	94	80	100	100	100	100	95	62
1200 mg on day 1 + 800 mg q10d	73	44	100	100	100	97	75	25
1200 mg q7d	99	94	100	100	100	100	99	87
1200 mg q10d	91	74	100	100	100	99	92	55
1200 mg q14d	65	38	100	100	99	94	68	21
1200 mg q21d	22	7	99	97	90	65	24	2
1200 mg day 1 + 1200 mg on day 8 + 1200 mg q14d	65	38	100	100	99	93	67	22

^a^: MIC_90_ for *E. faecium*; ^b^: MIC_90_ for *E. faecalis*; ^c^: MIC_90_ for *S. aureus* and coagulase-negative staphylococci; ^d^: EUCAST clinical breakpoint of *S. aureus*; ^e^: EUCAST clinical breakpoint and MIC_90_ for β-hemolytic streptococci. MIC: minimum inhibitory concentration; C_min_: minimum concentration; *f*C_min_: minimum free concentration; q7d: every 7 days; q10d: every 10 days; q14d: every 14 days; q21d: every 21 days.

## Data Availability

All data are contained within the paper.
