# Correction: Alarcia-Lacalle et al. Oritavancin Multiple Dosing for Complex Infections: A Pharmacokinetic/Pharmacodynamic Simulation Study. *Pharmaceutics* 2026, *18*, 628

**DOI:** 10.3390/pharmaceutics18080912

**Published:** 2026-07-24

**Authors:** Ana Alarcia-Lacalle, Miguel Ángel Morán-Rodríguez, Laura Morata, Arantxa Isla, Andrés Canut-Blasco, Alicia Rodríguez-Gascón

**Affiliations:** 1 Pharmacokinetic, Nanotechnology and Gene Therapy Group (PharmaNanoGene), Faculty of Pharmacy, Lascaray Research Centre, University of the Basque Country EHU, Paseo de la Universidad n° 7, 01006 Vitoria-Gasteiz, Spain; ana.alarcia@ehu.eus (A.A.-L.); arantxa.isla@ehu.eus (A.I.); 2 Infectious Disease Division, Araba University Hospital, Osakidetza Basque Health Service, Francisco Leandro de Viana Kalea, 01009 Vitoria-Gasteiz, Spain; miguelangel.moranrodriguez@osakidetza.eus; 3 Microbiology, Infectious Disease, Antimicrobial Agents, and Gene Therapy, Bioaraba Health Research Institute, Jose Atxotegi Kalea s/n, 01009 Vitoria-Gasteiz, Spain; andres.canutblasco@osakidetza.eus; 4 Department of Infectious Diseases, Hospital Clínic of Barcelona, University of Barcelona, Carrer de Villarroel, 170, 08036 Barcelona, Spain; lmorata@clinic.cat; 5 Microbiology Service, Araba University Hospital, Osakidetza Basque Health Service, Francisco Leandro de Viana Kalea, 01009 Vitoria-Gasteiz, Spain

## Error in Figure

In the original published paper [1], there was a mistake in “Figure 1” as published. In Figure 1, some graphs were inadvertently mismatched with their descriptive subtitles; this mistake occurred during the assembly of the final figure from the individual graphs. The authors state that their scientific conclusions are unaffected. The corrected Figure 1 appears below. This correction was approved by the Academic Editor. The original publication has also been updated.

## Figures and Tables

**Figure 1:**
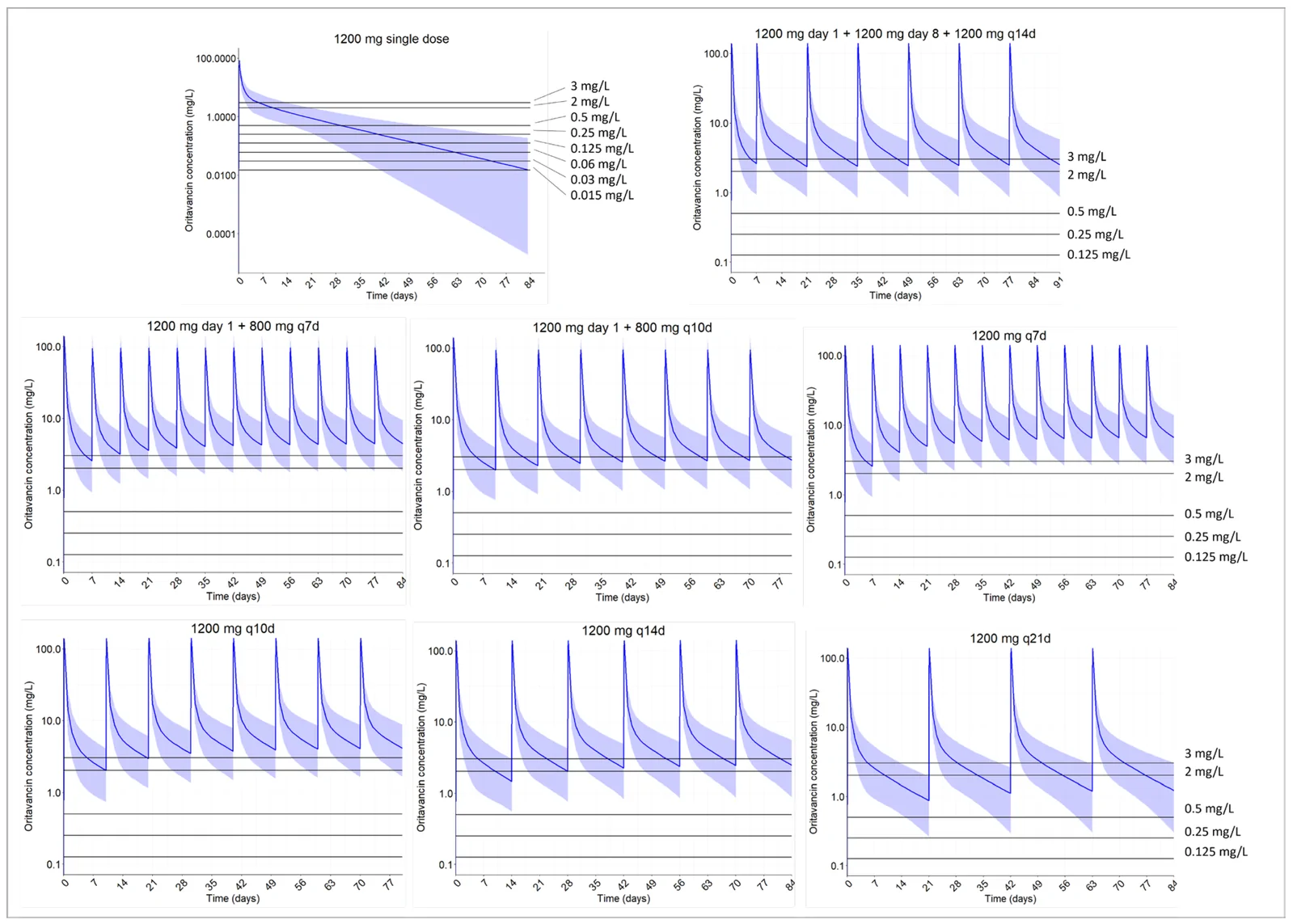
Simulated oritavancin plasma concentration–time profiles for different dosage regimens. The blue line represents the median of the simulated oritavancin plasma concentrations, and the shaded area represents the simulation-based 95% confidence interval for the median. Horizontal dotted lines indicate the concentration values used for the estimation of PK/PD target attainment (concentration thresholds of 2 mg/L and 3 mg/L, and representative MIC values).
